# Okadaic Acid: More than a Diarrheic Toxin

**DOI:** 10.3390/md11114328

**Published:** 2013-10-31

**Authors:** Vanessa Valdiglesias, María Verónica Prego-Faraldo, Eduardo Pásaro, Josefina Méndez, Blanca Laffon

**Affiliations:** 1 Toxicology Unit, Department of Psychobiology, University of A Coruña, A Coruña E15071, Spain; E-Mails: pspasaro@udc.es (E.P.); blaffon@udc.es (B.L.); 2 Department of Cellular and Molecular Biology, University of A Coruna, A Coruña E15071, Spain; E-Mails: veronica.prego@udc.es (M.V.P.-F.); fina@udc.es (J.M.)

**Keywords:** okadaic acid, diarrheic toxin, toxicity, phosphatase inhibition

## Abstract

Okadaic acid (OA) is one of the most frequent and worldwide distributed marine toxins. It is easily accumulated by shellfish, mainly bivalve mollusks and fish, and, subsequently, can be consumed by humans causing alimentary intoxications. OA is the main representative diarrheic shellfish poisoning (DSP) toxin and its ingestion induces gastrointestinal symptoms, although it is not considered lethal. At the molecular level, OA is a specific inhibitor of several types of serine/threonine protein phosphatases and a tumor promoter in animal carcinogenesis experiments. In the last few decades, the potential toxic effects of OA, beyond its role as a DSP toxin, have been investigated in a number of studies. Alterations in DNA and cellular components, as well as effects on immune and nervous system, and even on embryonic development, have been increasingly reported. In this manuscript, results from all these studies are compiled and reviewed to clarify the role of this toxin not only as a DSP inductor but also as cause of alterations at the cellular and molecular levels, and to highlight the relevance of biomonitoring its effects on human health. Despite further investigations are required to elucidate OA mechanisms of action, toxicokinetics, and harmful effects, there are enough evidences illustrating its toxicity, not related to DSP induction, and, consequently, supporting a revision of the current regulation on OA levels in food.

## 1. Introduction

Marine algal blooms, natural phenomena produced by the overgrowth of microscopic marine algae, have become a public health concern due to their increasing frequency and severity. About 300 phytoplanktonic species have the ability to cause these blooms, and a quarter of them are able to produce toxins, also called phycotoxins [1]. These phycotoxins are accumulated by fish and shellfish, mainly bivalve mollusks, although new vectors are continuously appearing in the literature [2], by direct filtration of the producer algal cells or by feeding on contaminated organisms. Human intoxications caused by phycotoxins occur worldwide through consumption of marine fishery products containing bioaccumulated toxins.

Okadaic acid (OA) and its analogs, the dinophysistoxins (DTXs), are lypophilic marine toxins produced by several phytoplanktonic species and responsible for the diarrheic shellfish poisoning (DSP) in humans. DSP toxins are one of the most relevant groups of the phytoplanktonic toxins since their presence produce not only health effects in human consumers but also severe economical losses [3]. OA, the main toxin representative of this group, was firstly described in 1981 from the black sponge *Halichondria okadai*, as well as from *H. melanodocia* [4,5]. It is produced principally by dinoflagellates of the genus *Prorocentrum* (mainly *P. lima*, but also *P. concavum*) and *Dynophysis* (mainly *D. acuta*, *D. acuminate*, and *D. fortii*) [6]. OA, like other phycotoxins, is accumulated by shellfish, mainly bivalve mollusks—such as mussels, scallops, oysters, or clams—and several fishes by eating the phytoplankton, and, subsequently, can be consumed by humans causing alimentary intoxications. The characteristics of these toxins are not changed by cooking or freezing and they do not modify the taste of the contaminated organisms, so they are very difficult to detect [7,8]. DPS toxins, OA, and DTXs, are widely distributed all over the world, but especially abundant in Europe, Japan, and South America [9,10], although new DSP episodes are continuously appearing (Canada, Mexico, India, Thailand, China, and Australia) [10,11]. The relationship of OA-related toxins with gastrointestinal symptoms was established a few decades ago. The first registered DSP episode after shellfish consumption occurred in 1961 in The Netherlands [12]. However, no relationship with the phycotoxins was established at that moment. It was in 1976 when the association between the frequent occurrence of gastroenteritis and the ingestion of phycotoxin-contaminated shellfish was proven for the first time [13]. Since then, a large number of DSP episodes have been documented worldwide. However, this number is believed to be even much higher as these episodes are not often well registered, since the acute symptoms are not always severe and frequently the intoxicated people do not require medical assistance.

It was early discovered that, together with provoking DSP syndrome, OA could cause inhibition of certain protein phosphatases (PP), particularly PP of type 1 (PP1) and 2A (PP2A) [14], but also other types including 4, 5, and 2B (PP4, PP5, PP2B) [15]. This property is not unique of OA; most OA group toxins have shown the ability to inhibit some PP to a greater or lesser extent. OA, DTX-1 and DTX-2 have all been demonstrated to be potent inhibitors of both PP1 and PP2A, with a significantly higher affinity for PP2A [16]. Still, there are some differences between them. Published data indicate that the affinity of PP2A for DTX-1 is 1.6-fold higher, and for DTX-2 is 2-fold lower than for OA [17]. However, other OA analogs such as 19-*epi*-okadaic acid or belizeanic acid were recently found to induce lower inhibitory potencies, mainly related to PP1 [18,19]. Particularly, a concentration of 150 nM of 19-*epi*-okadaic acid was required to induce the same neurotoxic effects observed at 0.5 nM OA or at 2.5 nM DTX-1 treatments [20]. In contrast, DTX-3 is inactive against these proteins if not hydrolyzed [21]. The PP inhibition leads to a dramatic increase of phosphorylation of a number of proteins resulting in important cell alterations, and this is the main reason why OA is an invaluable pharmacological tool in the study of cellular signaling [22]. In addition, because of this property, it was suspected that this toxin is much more than a simple diarrheic agent. Even nowadays, a number of cellular alterations related to OA’s ability to inhibit PP have been reported the mechanistic processes triggered by this toxin are not totally understood since not all the effects observed after OA exposure can be explained by this inhibition.

In the last few decades, the potential toxic effects of OA, beyond its role as a DSP toxin, have been investigated in several *in vitro* and *in vivo* studies. Alterations to DNA and cellular components, as well as effects on immune and nervous system, and even on embryonic development, have been increasingly reported. In addition, its potential role as a carcinogenic agent was pointed out by different authors. In this review, results from all these studies are compiled and revised in order to clarify the role of this toxin, not only as a DSP inductor, but also as cause of alterations at the cellular and molecular levels, and highlight the relevance of biomonitoring its effects on human health.

## 2. Acute Toxicity and DSP Symptomatology

The ingestion of OA contaminated shellfish results in DSP syndrome characterized by severe gastrointestinal symptoms, including diarrhea (92%), nausea (80%), vomiting (79%), abdominal pain (53%), and chills (10%) [23]. The intensity of these symptoms in humans depends upon the amount of toxin ingested. An intake of 40 μg of OA equivalents is the minimum dose required to produce DSP symptoms in human adults [24]. They appear within 30 min to 4 h after intake and continue for about three days, but they are not considered lethal and hospitalization is usually not required [8]. DSP is generally considered non-life-threatening, but complications may occur as a result of severe dehydration in some patients. To our knowledge, no information on possible longer term effects or effects related to repeated exposures is, thus far, available.

A median lethal dose (LD50) of 192 µg/kg level was established after intraperitoneal injection in mice [25], whereas the lowest observed adverse effect level (LOAEL) in mice, by acute oral administration, was deduced to be 75 µg/kg body weight [26]. Human data from Japan (eight people, age 10–68) indicate a LOAEL of 1.2 to 1.6 µg/kg body weight [27]. In a second study from Norway, 38 of 70 adults were affected with DSP at levels ranging from 1.0 to 1.5 µg/kg body weight [28]. Recently, based on the information of different studies, the Scientific Panel on Contaminants in the Food Chain concluded that the LOAEL for human illness is in the region of 50 µg OA equivalents/person [9].

At the tissue level, OA was early reported to induce long-lasting contraction of smooth muscle from human arteries and rabbit aorta [29]. As smooth muscle contraction is triggered by the phosphorylation of one of the small subunits of myosin, it was suggested that OA might activate myosin P-light chain kinase or inhibit a myosin P-light chain phosphatase, leading to the diarrheic effect. Other later authors proposed the OA-induced stimulation of the phosphorylation of proteins that control sodium secretion by intestinal cells as the cause of diarrhea [30,31]. After evaluating OA-fed mice at different times, Wang *et al.* [32] recently confirmed that OA remarkably inhibited the intestinal PP activity, recovering the normal levels within 6 to 24 h. However, they compared several protein profiles and concluded that OA toxicity in mouse intestines was complex and diverse, and that multiple proteins, other than PP, were involved in the diarrheic process. In addition to DSP symptoms, severe morphological alterations were observed in different rodent organs, especially intestine and liver, after OA administration [33,34]. These alterations include formation of blebs on the cell surface, pronounced changes of the cell appearance, desquamation of the degenerated epithelium from the lamina propria, and degeneration of absorptive epithelium and of endothelial lining cells.

The degree of damage was demonstrated to be dose-dependent but also the route of administration and animal species may be an important determinant of the organotrophicity of OA *in vivo*. For instance, intraperitoneal OA injection was observed to induce changes in rodent enterocytes within 15 min after administration, whereas intravenous injection of OA induced similar but less extensive changes in the same cells [35]. In case of oral OA administration, longer times or higher doses are needed to observe similar injuries in the animal organs and/or mortality rates than those observed after intraperitoneal administration [21]. Even in the same animals, OA administered by intragastric intubation was found to cause intestinal damage, diarrhea, and death, with a detectable effect on the liver, whereas, when administered intravenously, it induced little effect on intestinal function, but caused a rapid dissolution of hepatic bile canalicular actin sheaths, congestion of blood in the liver, hypotension, and death at high doses [36]. Regarding the influence of the animal species on the OA toxicity, rats were found to be more tolerant to OA than mice as intestinal injury occurred at 375 µg/kg b.w. in the first case, but at 200 µg/kg b.w. in the second one [37].

To the best of our knowledge, no toxicokinetic data regarding absorption, distribution, metabolism, or excretion of OA in humans are available thus far. Only a recent *in vitro* study proved that OA can be absorbed by human digestive cells and move into the bloodstream [38]. However, toxicokinetic studies in mice concluded that OA administered by different routes is well-absorbed and quickly distributed throughout the organism [33,34,39,40], even passing through the placental barrier of pregnant animals [41]. Particularly, five minutes after being orally administrated, OA was found systemically distributed, being detected in lung, liver, heart, kidney, and small and large intestine [33]. Twenty-four hours after oral exposure, OA was also present in different organs of the animals, showing the highest concentration in intestinal tissue and stomach [34]. Different injuries in the intestine were also found when the administration was intraperitoneal [39]. In addition, these animal studies also demonstrated accumulation of OA in specific organs, as well as slow body elimination, since OA was detected in mouse liver two weeks after its oral administration, and it was excreted in feces during at least four weeks [33].

## 3. OA beyond its Role as DSP Inductor

### 3.1. Cytotoxicity

As mentioned before, the primary known cellular targets of OA are several classes of protein serine/threonine phosphatases that play central roles in the regulation of essential cellular processes, including growth, division, death, and maintenance of cytoskeletal structure. The OA-induced phosphatase inhibition causes severe alterations on phosphorylation states of many cellular proteins that can eventually lead to the collapse of regulatory processes and a variety of cellular disruptions. Accordingly, exposure to OA, even at low concentrations, has been shown to induce alterations in a number of cellular events [36,42,43,44]. These toxic effects have been classically related to the inhibition of protein phosphatases; however, the cellular and molecular effects of this toxin are not always clearly explained by this inhibition, and the existence of other targets, different from phosphatases, cannot be excluded [45]. In fact, the existence of OA binding proteins other than phosphatases was already demonstrated in several marine organisms [46,47]. This potential existence of OA non-phosphatase targets would explain the conflicting reports in the literature on OA cytotoxic effects, mainly, both, apoptosis and cell proliferation, in many cell types. In brief, little is known about the molecular mechanisms and the components involved in the cellular responses induced by OA beyond its role as phosphatase inhibitor and a re-evaluation of the mechanism of toxicity of this compound was already suggested [48].

The most extensively reported cytotoxic effect of OA is apoptosis induction. OA is known to cause growth inhibition or apoptosis in many, but not all, cell types including intestinal cells, neuronal cells, hepatic cells, lung cells, blood cells, *etc.* [44,49,50]. The mechanisms involved in this process include alterations in the expression of specific genes, decrease in the mitochondria membrane potential, activation of multiple caspase isoforms, cytochrome *c* release from the mitochondrial intermembrane space to the cytosol, inhibition of protein synthesis, and cytoskeletal disruption [49,50,51,52,53,54,55]. It seems that all these events are directly related to OA ability of inhibiting the phosphatase activity. Indeed, studies employing OA tetraacetate (and OA derivative without inhibitory activity of protein phosphatases), instead of OA for cell treatments, did not observe an increase in the apoptosis rate [56,57]. Nevertheless, OA was also reported to decrease the apoptosis induced by other, different compounds such as 1-methyl-4-phenylpyridinium ion [58] or glucocorticoids [59]. Ao *et al.* [51] exposed BALB/c 3T3 cells to OA and observed that numerous genes associated with cell proliferation and cell cycle progression were down-regulated at early and/or middle time points after treatment. These results suggest that multiple molecular pathways are involved in OA-induced proliferation inhibition and apoptosis.

One of the OA main cellular targets is the cytoskeleton. Alterations to the cytoskeleton are highly relevant since they are the preliminary step of many other damaging cellular events. Several cytoskeletal alterations have been reported in different cell systems after OA treatment. It was described to induce noticeable morphological changes, destabilization of microtubules, cell rounding, loss of stabilization of focal adhesions with consequent loss of cytoskeletal organization, and loss of barrier properties in different cell types [44,60,61,62]. It was previously suggested that these alterations could be due to different mechanisms, such as disruption of F-actin and/or hyperphosphorylation and activation of kinases that stimulate tight junction disassembly [50,63], alterations in the expression levels of relevant genes involved in the maintenance of the cell structure [64], or general activation of cell signaling pathways that induce breakdown of the cortical actin cytoskeleton and cell detachment [65]. In addition, as most of these morphological changes are typical of apoptotic processes, these two types of cytotoxic events may be highly related in OA exposed cells.

A number of studies reported OA-induced cell cycle alterations, mainly mitotic arrest and premature chromosome condensation, in different cell types, including leukemia cells [66,67], intestinal colon cells [43], lymphocytes [68], fibroblasts [69], and neuronal cells [44]. These effects on cell cycle can be due to the absence of dephosphorylation control of some kinases, as a consequence of protein phosphatases inhibition caused by OA. This imbalance would lead to an increase in proliferation, aberrant mitosis, or growth arrest, depending on the cell type. In addition, PP2A activity appears to be required for the metaphase–anaphase transition [67], which would explain the mitotic arrest found in OA-treated cells.

Together with apoptosis induction, cytoskeleton disruption, or cell cycle alterations, OA was also shown to induce oxidative stress in a number of *in vitro* and *in vivo* studies [70,71,72], and to affect different catabolic and anabolic pathways—including glycolysis, lipolysis, and gluconeogenesis—in several cell types [15,53]. Besides, the expression of different metabolism-related genes was found to be altered after OA exposure [73].

However, the cytotoxic effects induced by OA vary very considerably in different cell types. For instance, Rubiolo *et al.* [74] studied the cytotoxic effects of OA in two hepatic cell lines, namely HepG2 and Clone 9, and found dissimilar effects on the cell cycle for both types. OA arrested the cell cycle of Clone 9 cells in G_2_/M inducing aberrant mitosis, whereas arrested HepG2 cell cycle in G_0_/G_1_. The effect of the toxin on gene expression of cyclins and cyclin-dependent kinases was also different for both cell lines: increase in expression of cyclins A, B, and D in Clone 9 cells but decrease in cyclins A and B in HepG2 cells. Furthermore, Valdiglesias *et al.* [44] reported a clear cell cycle arrest at the S phase in neuronal cells exposed to OA, but no such arrest was observed in hepatic cells or lymphocytes under the same exposure conditions.

The exposure dose can also account for the divergent results concerning the OA cytotoxic effects. OA was demonstrated to have a concentration dependent effect on cell cycle and apoptosis induction. Thus, Ishida *et al.* [66] found different cell cycle arrest in myeloid leukemic cells, either at G_1_/S phase or at G_2_/M phase, depending on the OA dose. Recently, Fieber *et al.* [75] also observed that OA stimulates genes involved in the cell cycle progress at low concentrations whereas it promotes apoptosis at high concentrations. These results support the previous ones in which OA was found to induce mitotic arrest, followed by apoptosis in a synchronized manner [67].

### 3.2. Neurotoxicity

Despite not being classified as a neurotoxin, an increasing number of studies have reported the neurotoxic effects of OA, generally related to neuronal apoptosis, protein tau hyperphosphorylation, and morphological alterations, on both neuronal cells and animal systems [76,77,78]. OA, at concentrations ranging 5–100 nM for 12 or 24 h, was found to induce apoptosis in TR14 and NT2-N human neuroblastoma cells [77], mouse neuroblastoma cells [79], and rat cerebellum neurons [80,81], and at 1000 nM in SHSY5Y [44] human neuroblastoma cells. Furthermore, OA induced a number of cellular effects that, if not restored, can eventually lead to neural cell death too. Among them, it forced differentiated neuronal cells into the mitotic cycle [77], caused changes in microtubule associated proteins and neuronal cytoskeleton of cultured cortical neurons [76], and induced disintegration of neurites and swelling of cell bodies in cultured cerebellum neurons [20].

The phosphorylation and accumulation of tau protein was observed after OA treatment in several neural cell types including mouse [82] and human [83] neuroblastoma cells, rat cortical neurons [84], and neuronal cultures derived from cerebral cortex of early postnatal rats [85]. Phosphorylated tau was also found to be increased in adult rats treated with OA by direct microinjection into the lateral dorsal hippocampus area [86] or by chronic intraventricular injection into brain [87]. Tau protein is responsible, among others, for microtubule stabilization, maintenance of long-term potentiation, learning and memory, and its abnormally phosphorylated form is considered one of the pathological hallmarks of Alzheimer’s disease [88]. Accordingly, OA is currently employed as a useful tool to screen potential drugs for prevention and treatment of Alzheimer’s disease and other dementia forms [70,83,89,90].

The OA-induced cytoskeleton disruption previously described in this review becomes especially relevant in the case of nervous system cells given the significance of cytoskeletal organization in several key neural processes such as neurite outgrowth [91], synaptogenesis [92], structural polarity and neuronal shape [93], axonal transport [94], and neurotransmitter release [95]. In addition, cell shape and structural polarity are frequently lost in neurodegenerative diseases and neural aging [96,97]. Together with neuronal morphological alterations, OA was also found to modify the expression of genes related to cytoskeleton and neurotransmission in neuronal cells [64].

A low number of studies were carried out *in vivo* to evaluate behavioral changes induced by OA. After being directly administered into the dorsal rat hippocampus, it was observed to induce hyperexcitability and neuronal stress [98], deficits in spatial memory [99], cognitive deficits [86], and astroglial alterations and spatial cognitive deficits, even 12 days after OA exposure [70]. Subsequent dose- and time-dependent neurodegeneration was observed in all cases. In addition, a chronic OA injection into rat brain ventricles for up to eight weeks generated a neuronal protein redistribution that led to severe memory impairment [87]. This memory-impairment associated to OA exposure was also observed in other rodent studies [100,101]. In addition, Kamat *et al.* [101] found that the impairment was related to an increased expression of proinflammatory cytokines and total nitrite in several brain regions, indicating that neuroinflammation may play a vital role in OA-induced memory impairment. The results of these animal studies support the use of OA as an emerging tool for research in Alzheimer’s disease and other dementia forms, as some of the Alzheimer’s disease-like neuropathological signs may be induced in rats after chronic exposure to OA. In this regard, Kamat *et al.* [101] evaluated the effect of antidementia drugs, such as donepezil and memantine, on OA-induced neurotoxic alterations in rats. These drugs substantiated protection against memory impairment, confirming that OA neurotoxicity can be clinically prevented at least in part by using antidementia drugs.

### 3.3. Immunotoxicity

Although studies in mice have pointed out the immune system as one of the targets of different marine algal toxins [33,102,103], very little is known about the immunotoxic potential of OA.

*In vitro*, OA was early reported to produce important alterations on interleukin-1 (IL-1) production by human peripheral monocytes with stimulation at low concentration (0.05 µg/mL), a marked depression at medium concentrations (0.1–1.0 µg/mL) and cell death at higher levels [104]. In addition, the suppressive effect of OA on IL-1 was readily reversed by specific monoclonal anti-OA antibody. Further studies confirmed the OA-induced modifications on IL-1 production [105] and even modulation of IL-1 gene expression [106]. Interleukin 8 (IL-8) production, stimulated in most cell types under a variety of inflammatory stimuli, was also reported to be dramatically increased (up to 200-fold) in HL-60 cells treated with OA [107]. In this study, OA induced an important stimulation of IL-8 production at both transcriptional and post-transcriptional levels. A recent *in vitro* study employing mouse T lymphocytes also reported that low concentrations of OA (5 nM), within the range previously detected in mice bloodstream after oral administration, induced down-regulation of T cell receptor (TCR) expression levels in these cells, compromising the T cell activation and, consequently, the immune response [108].

OA effects on immune system of animals and humans have been poorly studied. In a study employing mice orally fed 17.8 µg/kg OA, different immunotoxic effects were observed including thymus morpho-functional modifications and atrophy, depletion in the lymphoid compartment, and angiogenesis [102]. Results of this study suggested that low oral doses of OA are able to induce immunostimulation and systemic immunotoxicity. Furthermore, an inflammatory cell response was activated in these animals by the recruitment of granulocytes, a higher number of active macrophages and increased immunoreactivity to cytokines. Recently, Kamat *et al.* [101] demonstrated that OA, after intracerebroventricular administration, caused memory impairment in rats and neuroinflammatory changes, including increased expression of proinflammatory cytokine tumor necrosis factor alpha (TNF-α) and interleukin 1 beta (IL-1β) in the hippocampus and cortex brain regions of these animals. Release of TNF-α and IL-1β cytokines initiates inflammatory cascades and are considered as markers of inflammation in peripheral tissue, as well as in the brain [109].

Despite these few works, the immunotoxic OA effects and its action mechanisms on the immune system remain unclear and further investigations on this regard are required, especially to define the immunotoxicity potential of OA on humans.

### 3.4. Embryotoxicity

There is no evidence of reproductive or developmental toxicity of OA on humans in the literature; however, several studies performed in animals, usually fish and frogs, have reported embryotoxic effects of this toxin. It was early found that microinjection of OA in frog (*Xenopus laevis*) oocytes and starfishes (*Marthasterias glacialis* and *Astropecten aranciacus*) induced the meiotic maturation and the activation of the mitosis promoter factor [110,111]. More recently, Escoffier *et al.* [112] incubated medaka fish (*Oryzias latipes*) embryos in a medium containing OA and observed retardation of embryo development and dose-dependent reduction in survival rate, achieving 100% mortality at 0.75–1 µg/mL. Another work performed with frog (*X. laevis*) embryos in the same year confirmed that OA treatment induces embryo malformations, increase in mortality and also delayed growth in this species [113]. In that study, *X. laevis* embryos were treated with 0.1, 1, and 10 nM OA and the Frog Embryo Teratogenesis Assay-Xenopus (FETAX assay), a powerful and flexible bioassay for developmental toxicants, was performed. Mortality was observed after two-day incubation with 1 and 10 nM OA, whereas delayed growth and embryo malformation was also detected at 0.1 nM OA. Franchini *et al.* [114] examined the effects of OA on some genes involved in the neural and muscular specification and patterning of *X. laevis*. They performed the analysis at different stages of embryonic and larval development and found that this toxin induced important alterations in the expression of several development-related genes. In addition, the response to OA resulted stage-dependent, with the embryonic development stage more sensitive to the toxin than the larval stages.

Together with these studies, OA was proven by two independent bioassays to exhibit a weak teratogenicity on cultured murine embryonic cells even at a 5 nM concentration [115], to increase the meiotic resumption and maturation rates of canine oocytes [116], to affect meiotic resumption of blue fox oocytes *in vitro* [117], and to resume meiosis with fast kinetics of germinal vesicle breakdown through the MEK-MAPK pathway in incompetent growing mouse oocytes [118]. In addition, OA was found to induce premature chromosome condensation, meiosis resumption, pronucleus breakdown, inhibition of spindle organization, and microtubule assembly suppression by sperm centrosomes in porcine oocytes and fertilized eggs [119].

The OA-induced effects observed in all these *in vitro* and *in vivo* studies might involve an irreversible damage to embryonic development, therefore there is an imperative need to assess the risk of orally consumed OA on human unborn life.

### 3.5. Carcinogenicity and Genotoxicity

#### 3.5.1. Genotoxicity and Effects on DNA Repair

OA is a well-established genotoxic agent since damage on the genetic material was described in a number of human and animal cell types after OA exposure [120,121,122,123,124]. This damage includes a variety of DNA lesions such as micronuclei formation, oxidative DNA damage, sister chromatid exchanges, 8-hydroxy-deoxyguanine adducts, minisatellite mutations, and DNA strand breaks.

However, the nature of genotoxic OA effects is not entirely defined since data reported are often contradictory. Some studies, employing different cell types, indicate that OA is an aneugenic agent [120,121,122,123,124,125]. Carvalho *et al.* [120], for example, carried out the cytokinesis-block micronucleus assay in combination with fluorescence *in situ* hybridization (FISH) on Caco2 cells in order to discriminate between clastogenicity and aneugenicity, and they found that OA caused whole chromosome loss suggesting a specific aneugenic potential. The same effect was observed in CHO-K1 cells by employing the same technique [125], and in fibroblasts, keratinocytes, leukocytes, and lymphoblastoid cells by other different approaches [121,122,123,124]. However, other authors propose double strand breaks (DSB) production as the genotoxicity mechanism of action for this toxin [43,124]. There are also works indicating that OA needs to be metabolically activated to exert its mutagenic action [125,126], contrasting with other investigations demonstrating a predominantly direct effect [124,127]. Furthermore, despite not inducing mutations in bacterial assays either in the absence or in the presence of metabolic activation, OA showed strongly mutagenic properties in Chinese hamster lung cells without metabolic activation; its mutation frequency was comparable to 2-amino-*N*6-hydroxylamine [128].

It seems that this controversy might be based on the fact that OA genotoxic effects are highly dependent on cell type and experimental conditions. Souid-Mensi *et al.* [42], and later Valdiglesias *et al.* [124], evaluated the effects of OA on different cell types and found notable differences in the cell responses to the toxin associated with the cell type, the concentration and the exposure time, confirming that these factors would be behind the controversial OA genotoxicity data found in the literature.

Moreover, the OA genotoxic potential is not only restricted to a direct induction of damage in the genetic material, but it can also alter the repair of the DNA damage induced by other genotoxic compounds [129]. The DNA repair system has been recognized as one of the most important cellular defense mechanisms responsible for the integrity of DNA. Decreased DNA repair ability is exhibited in various clinical conditions and associated with increased frequency of carcinogenesis, since unrepaired mistakes lead to an accumulation of aberrations in the genome that culminate in the genetic instability typical of many malignancies and other pathologies [130]. A limited number of previous studies were performed in order to determine the possible interference of OA with DNA repair mechanisms. The phosphatases inhibition caused by OA has been suggested to be able to slightly inhibit DNA repair in a dose-dependent manner [131] and to affect non-homologous end-joining DSB repair [132]. In addition, Valdiglesias *et al.* [129] used the DNA repair competence assay to evaluate the effects of OA on DNA repair of different human cell lines, namely hepatocytes, neuronal cells, and lymphocytes, and they concluded that OA disrupts the DNA repair processes to a different extent in the three cell types, being hepatocytes the most sensitive to its effects. However, Le Hegarat *et al.* [133] reported that OA does not interact with the DNA repair process in rat hepatocytes using unscheduled DNA synthesis.

On the basis of these results, it seems that OA is able to induce damage in the genetic material and also to alter the machinery responsible for its repair. As alterations in DNA repair mechanisms may affect the susceptibility of individuals exposed to a particular mutagen, and deficiencies in these mechanisms have been linked to the presence of a large number of diseases and cancer [134,135], OA exposure may increase the susceptibility to other genotoxic agents and, thus, the risk of adverse health outcomes.

#### 3.5.2. Tumor Promotion

At the moment, OA has not been classified by the International Agency for Research on Cancer (IARC) regarding its carcinogenicity potential. Nevertheless, OA showed very potent tumor promoting activity in two-stage carcinogenesis experiments involving mouse skin and mucosa of the rat glandular stomach after dermal and oral administration, respectively [136,137]. It has been reported that disruption of cellular processes regulating the transitions from G_0_ to G_1_ to S-phase is an important early step in tumor promotion by OA [138]. Furthermore, the effects of OA as tumor promoter were suggested to be mediated in part by the transcription factor AP-1 [139], and an NH_2_-terminal deletion of c-jun, called TAM-67, blocks OA-induced AP-1 activation and protected from tumorigenesis promoted by OA [140]. In addition, this toxin was observed to induce several genotoxic and cytotoxic effects including DNA strand breaks, DNA adducts, cell cycle alterations, apoptosis, oxidative damage, DNA repair alterations, and micronuclei induction (reviewed in previous sections). All these disruptions may, if they are inaccurately repaired or left unrepaired, cause genomic instability that leads to severe pathologies including cancer. In addition, the OA-induced immunostimulation and systemic immunotoxicity previously reported in mice [98] can be also indicative of tumorigenic properties of this toxin.

It was also reported that other marine toxins, different from OA, can also act as specific protein phosphatases (mainly PP1 and PP2A) inhibitors [141]. They are called OA class tumor promoters and were demonstrated to be able to cause skin, stomach, and liver tumors in animals (reviewed in [141]). This has led some authors to suggest a new concept of tumor promotion: the okadaic acid pathway. Further research is still required to shed light on the underlying mechanisms of OA- and other OA class tumor promoters-related tumorigenesis.

#### 3.5.3. Carcinogenicity

No human epidemiological studies with quantitative data on effects of OA exposure beyond the DSP syndrome have been reported so far. However, two human population studies, not based on experimental data but on information obtained by means of questionnaires, indicated a significant association between diarrheic intoxication (DSP) and several types of cancer, specifically esophagus, stomach, colon, pancreas, and liver cancers [142,143]. These results together with the previous findings in animals, have led some authors to suggest that a continued OA exposure could induce different types of cancer in humans [142,144]. Recently, Valdiglesias *et al.* [145] evaluated the expression levels of several genes directly or indirectly related to cancer initiation or progression, in human neuronal cells treated with OA. Results obtained showed important alterations in the expression patterns of a number of genes at one or more treatment times (3, 6, and 24 h). Although more exhaustive studies are required before drawing any final conclusion because of the complexity of the cancer processes, this study supports the hypothesis previously proposed by Cordier *et al.* [142], and Manerio *et al.* [144], providing a possible molecular explanation of the potential relationship between the consumption of OA-contaminated shellfish and the incidence of different cancers in humans.

## 4. Conclusions and Perspectives

OA is a marine toxin that can be easily accumulated by several shellfish and fish species causing frequent human food poisonings by the consumption of contaminated organisms. It is a highly interesting compound as it can be used as a very useful tool in the study of cellular signaling due to its ability to inhibit selectively the protein phosphatase activity. In the last few years, there has been an increasing number of toxicological studies reporting OA toxic effects at different levels, including citotoxicity, genotoxicity, immunotoxicity, embryotoxicity, neurotoxicity, and carcinogenicity. It was thereby demonstrated that the toxicity of this compound is not limited to the acute hazards related to its diarrheic potential, but also involves a significant risk at the cellular, molecular, and genetic levels. Furthermore, its mechanism of action appears to be quite complex and, although it seems that most effects are associated with the inhibition of protein phosphatases and its consequences, not all the effects may be explained by this inhibition and other possible molecular or cellular targets cannot be ruled out.

There is no reported data on chronic or subchronic effects of OA in humans. However, on the basis of a number of *in vitro* and *in vivo* studies, chronic exposure to DSP is suspected to be related to tumor formation in the digestive system since all the disruptions produced, if not correctly repaired, cause genomic instability that may lead to severe pathologies including cancer.

Despite all the works reported on OA effects in last few decades, there are still important gaps in the knowledge of the action mechanism of this toxin: the lack of information concerning immunotoxicity, embryotoxicity, and teratogenicity, unawareness on human toxicogenetics, absence of studies evaluating the chronic or subchronic effects of OA on animal species or humans, controversial results regarding genotoxicity and cytotoxicity, lack of identification of potential non-phosphatase targets, absence of human population studies, *etc.* Consequently, further investigations are required to elucidate the OA toxicity on humans and completely understand the risk of exposure to this toxin, not just as DPS inductor but also as a potentially mutagenic and carcinogenic compound.

The current European regulation (Regulation (EC) No. 853/2004, 29 April 2004) [146] on the level of DSP toxins in shellfish for human consumption exclusively focuses on reduction of the gastrointestinal symptoms. This regulation establishes a maximum permitted level of 160 µg of OA equivalent per kg. This means that small quantities of OA may be present in mollusks that have passed legal controls before their marketing, and therefore regular consumers might be chronically exposed to this toxin. Thus, a decrease of this level (from 160 µg/kg to 45 µg/kg) was already proposed in a recent report by the European Food Safety Authority (EFSA), based on acute effects on consumers [9]. In view of all data reviewed in this manuscript, and despite the important gaps in the knowledge of OA behavior, there are enough evidences illustrating its toxicity beyond its role as a DSP toxin and consequently supporting the reduction of the current EU limit.
